# Prevalence and resistance profile of bacteria isolated from wound infections among a group of patients in upper Egypt: a descriptive cross-sectional study

**DOI:** 10.1186/s13104-023-06379-y

**Published:** 2023-06-19

**Authors:** Eman Farouk Ahmed, Asia Helmi Rasmi, Abdou M. A. Darwish, Gamal Fadl Mahmoud Gad

**Affiliations:** 1 Assistant Professor of Microbiology and Immunology, Microbiology and Immunology Department, Faculty of Pharmacy, Sohag University, 82524 Province, Sohag, Egypt; 2 Teaching assistant of Microbiology and Immunology, Microbiology and Immunology Department, Faculty of Pharmacy, Deraya University, Elminya, Egypt; 3 Professor of Plastic and Reconstructive Surgery, Plastic and Reconstructive Surgery Department, Faculty of Medicine, Minia University, Elminya, Egypt; 4 Professor at Microbiology and Immunology, Microbiology and Immunology Department, Faculty of Pharmacy, Minia University, Elminya, Egypt

**Keywords:** Egypt, Prevalence, Wound infection, *S. aureus*, *P. aeruginosa*, MRSA, MDR

## Abstract

**Aim:**

This cross-sectional survey aimed to identify aerobic bacteria, antimicrobial resistance, and multi-drug resistance profiles of bacteria isolated from different wound infections among a group of Egyptian patients.

**Results:**

Of 120 positive samples, 170 isolates were identified. Polymicrobial infections were determined in 55% of samples. The dominant Gram-positive isolated strains were *Staphylococcus aureus*, especially from wound infections because of accidents (71.8%). Piperacillin, methicillin, ampicillin/sulbactam, and amoxicillin/clavulanic acid were all highly resistant to *S. aureus* and Coagulase-negative Staphylococci. The prevalence of methicillin-resistant *S. aureus* in wound infections was 89.9%. *S. aureus* showed superior sensitivity to vancomycin (85.3%) and linezolid (81.3%). The highest prevalence of Gram-negative isolates was for *Pseudomonas aeruginosa* (40%), which was highly sensitive to ciprofloxacin (79.2%) and highly resistant to levofloxacin (83.3%). Several isolates revealed a multi-drug resistance profile (52.4%). The overall MDR rate of Gram-positive and Gram-negative isolates were 50% and 54.9%, respectively.

**Conclusion:**

The prevalence of MRSA isolated from various wound infections and MDR is a warning issue in Upper Egypt. It should implement a health education strategy and hygiene measures to prevent the spread of wound infection-causing organisms in the community.

## Introduction

Skin provides a good medium for pathologic bacteria to proliferate. Subsequently, the chance of a skin wound being infected is boosted, and interferes with the healing process [1]. The origin of wounds varies, from acute postsurgical wounds (i.e., surgical site infections), post-traumatic wounds (i.e., wounds following an accident or burns), or chronic wounds such as those associated with diabetes mellitus (e.g. diabetic foot ulcers) [2].

The rate of MDR bacteria elucidates a worldwide increase as announced by the Centers for Disease Control and Prevention (CDC) [3]. This phenomenon has negative implications for the healthcare system and increases the threat of antibiotic failure, which raises the mortality rates [4]. More than 90% of *S. aureus* are resistant to penicillin, and that remains a global issue [5]. Despite that, MRSA strains have the tendency to expand quickly within a health facility via colonized or infected patients or health professionals, as well as contaminated sites within the facility [6]. Normally, antibiotic resistance gradually emerges. The injudicious administration of broad-spectrum topical and systemic antibiotics hastens the emergence of resistant bacteria [7]. Unfortunately, in developing countries, the unwise use problem of antibiotics is exacerbated by the absence of strict precautions to dispense the antibiotics without a medical prescription [8].

Regardless the type of wound infection, it is important to monitor the dynamic changes in the prevalence of sensitivity profiles and MDR profiles over time. This is important in detecting a structured therapeutic strategy to prevent microbial proliferation while avoiding side effects [9]. Regarding the MDR of bacteria isolated from different kinds of wound infection in Upper Egypt, the current survey was conducted to identify aerobic bacteria isolated from a group of Egyptian patients with different types of wounds and burn infections attend Minia University Hospital. Furthermore, to detect the antimicrobial resistance profile and MDR profile of different bacterial isolates.

## Materials and methods

### Design and setting

The study designed as a cross-sectional design that has been carried out from November 2019 to September 2021. The wound samples were collected from the patients who attended the Department of Plastic and Reconstructive Surgery, Minia University Hospital (MUH). The hospital provides its services to a geographical area of approximately 6 million people in Upper Egypt. The hospital included 330 beds.

### Demographic data of the study population

The study included males and females aged 1 month to 60 years. Wound infection is suspected if a wound was not healing well, getting bigger, and exudation of pus or fluids. Samples were obtained from various wound types including burn wounds, surgical wounds from different anatomical sites, and abscesses. Specimens were properly labeled, indicating the source, gender, and age of patients.

### Sample size

A total of 146 randomly selected wound swabs were collected from MUH. Wound infection is suspected when a wound is not healing properly, grows in size, or exudes pus or fluids. The specimens were collected on sterile cotton swabs and the wounds were cleaned before collection to avoid surface contamination. Swabs are transferred immediately, within 2 h to the laboratory. In the laboratory, the specimens were registered, and swabs were cultured, streaked, and incubated at 37 °C for 24 h. After incubation, plates were checked for bacterial growth. Plates with negative bacterial growth were additionally incubated for another 24 h.

### Ethical consideration

The hospital collects samples from different clinical sources on a daily basis from patients and sends them to the hospital’s laboratories for analysis. Samples were collected from hospital laboratories without dealing with patients directly. Upon getting permission from the Head of Plastic and Reconstructive Surgery Department, Faculty of Medicine, Minia University, the collection of laboratory samples has begun.

### Isolation and identification of wound bacterial isolates

The microorganisms were identified by Gram stain, culturing, biochemical reactions, and motility testing as per standard guidelines [10]. Culture plates of Nutrient agar, Muller Hinton agar, nutrient broth, Cetrmide agar, Mannitol salt agar (MSA), Brain heart infusion broth, Triple sugar iron (TSI) agar and DNase agar (Oxoid, England). Simmon citrate agar (CONDA). MacConkey agar, Eosin methylene blue, and Sulphide indo motility test (SIM), all produced by Himedia Laboratories, India. All media were prepared according to the instructions of the manufacturers. The media were sterilized by autoclaving at 121ºC for 15 min. Subcultures were then made into plates of nutrient agar, MSA, and MacConkey agar and incubated for another 24 h. The primary identification of the bacterial isolates was based on colonial appearance, pigmentation, morphology, and Gram staining characteristics using a light microscope. Biochemical tests were performed to identify the isolates. Biochemical tests were the standard catalase test, coagulase (tube and slide) test, DNase test, triple sugar iron test, Simmon citrate test, Indole, and sulphide production motility. Colonies were maintained by storing them at -80˚C in stocks with 2.5 M glycerol.

### Antibiotic sensitivity testing

Antimicrobial sensitivity was determined by the Kirby Bauer agar disc diffusion method. A small inoculum of each pure bacterial isolate was emulsified in 2 mL sterile normal saline. The turbidity of the cell suspension was adjusted to correspond to 0.5 MCfarland standard (1.5 × 10^8^ CFU/mL). The inoculum was dispensed on the surface of Mueller-Hinton agar plate and ramified with a sterile metallic wire loop and the plates were allowed to dry for 3–5 min. Antibiotic discs were used with the following concentrations: linezolid (30 µg, Bioanalyse limited -Turkey), tetracycline (30 µg, Himedia India), chloramphenicol (30 µg, Bioanalyse limited -Turkey), rifampin (5 µg, Himedia India), piperacillin (100 µg, Bioanalyse limited -Turkey), amoxicillin/ clavulanic acid (30 µg, Bioanalyse limited -Turkey), ampicillin/ sulbactam (20 µg, Bioanalyse limited -Turkey), levofloxacin (5 µg, Himedia India), gentamicin (10 µg, Himedia India), vancomycin (30 µg, Himedia India), cefoxitin (30 µg, Sigma USA), ciprofloxacin (5 µg, Bioanalyse limited -Turkey), cefuillin/tazobactam (75/10 µg, Himedia India), cefuroxime (30 µg, Himedia India), ceftazidime( 30 µg, Himedia India ), tigecycline( 15 µg, Himedia India), cefazolin (30 µg, Himedia India ), trimethoprim-sulfamethoxazole (1.25/23.75 µg, Himedia India), and imipinem (10 µg, Himedia India). Antibiotic discs were applied on the surface of the plates at least 15 mm apart from the edges of the plates to prevent overlapping of inhibition zones. The plates were incubated at 37^o^C for 24 h and the diameters of zones of inhibition were measured in mm and the results are compared to those from the Clinical Laboratory Standard Institute (CLSI) **(CLSI, 2018)**. *S. aureus* isolates were considered MRSA when the diameter of inhibition zone of cefoxitin disc is ≤ 21 mm according to CLSI **(CLSI, 2018).**

### Statistical analysis

Statistical analyses were performed using the chi-square or Fisher’s exact test using SPSS software version 16 (SPSS, Inc., Chicago, IL, USA). The results were considered statistically significant when the P value was less than 0.05.

## Results

### Participant’s demographic characteristics and wound samples

**Fig. 1:**
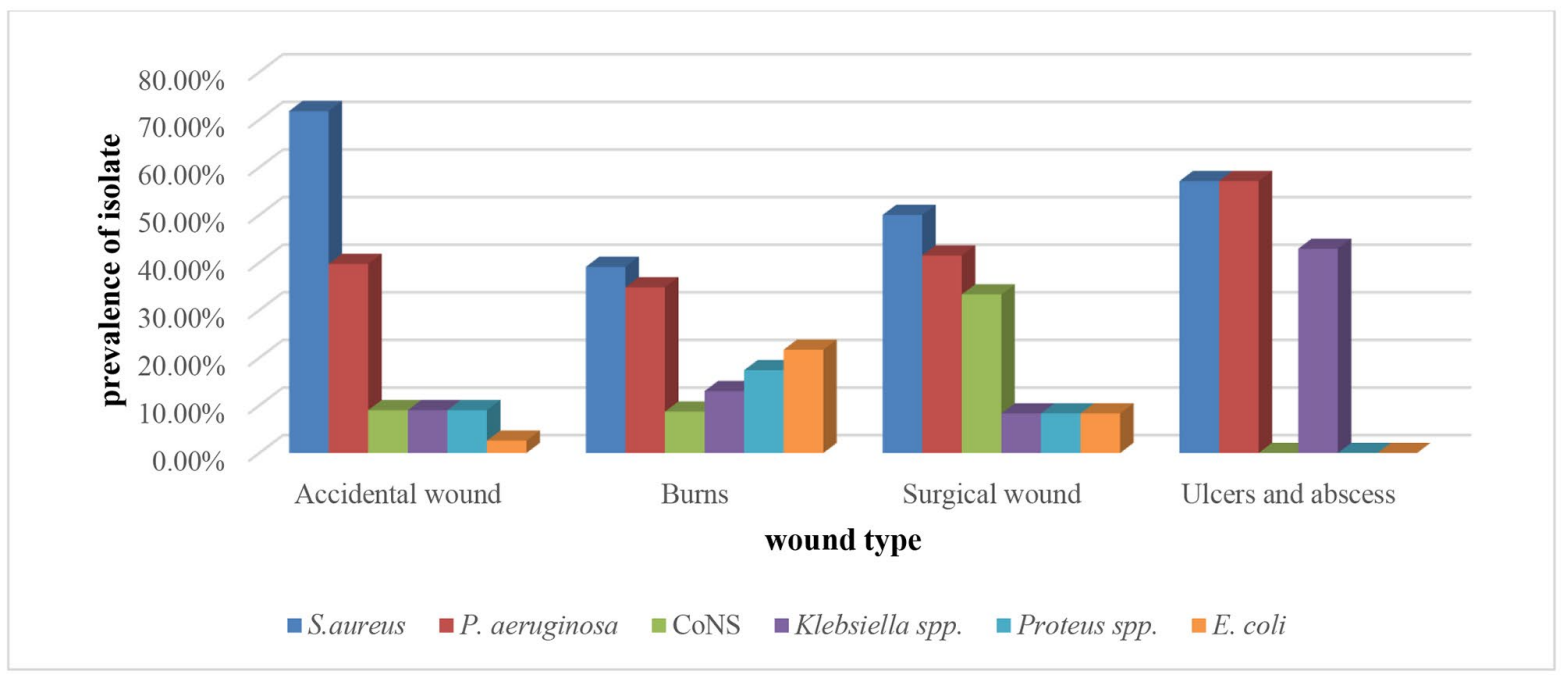
Prevalence of isolates from patients suffering from different types of wound infections. % was calculated out of number of samples obtained from a certain kind of wound

Of 146 samples, bacterial growth was evident in 120 (82.2%) samples, and 26 cultures (17.8%) were clear. The majority of participants were males, representing 73.5% (n = 88) and the rest was females represented 26.7% (n = 32). The ages of the patients ranged from 1 month to 60 years, with an average of 25.28 ± 16.21. The frequencies of samples isolated from different wound infections were shown in Fig. 1.

### Bacterial isolate identification and distribution among wound infections

**Fig. 2:**
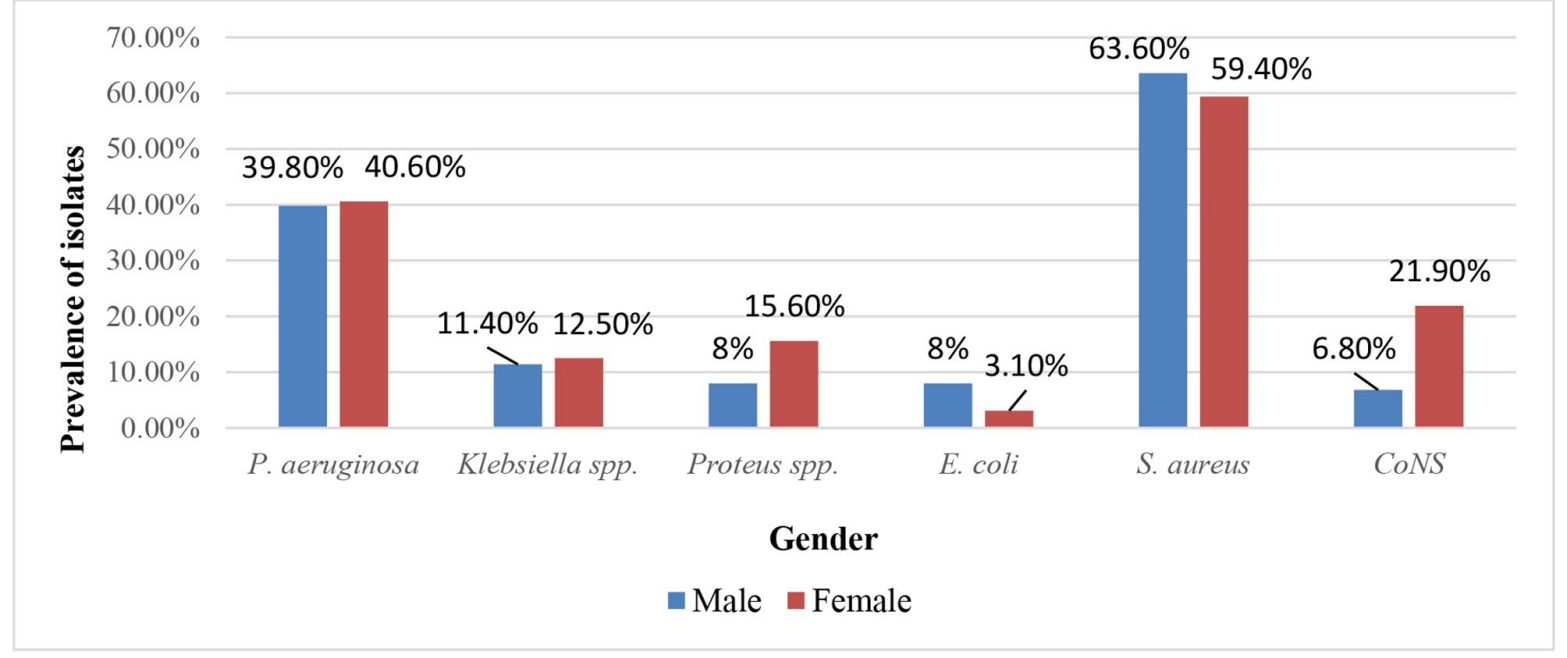
Prevalence of bacteria isolated from different types of wound infections in relation to gender. % of bacterial isolates was calculated out of total number of males or females

Out of 120 samples, 170 isolates were identified. Poly-microbial infections were determined in 66 (55%) samples, while mono-microbial infections were detected in 54 (45%) samples. There were 88 Gram-positive isolates (51.8%) and 82 Gram-negative isolates (48.2%). The dominant strains isolated from all types of wound infections were *S. aureus* (62.5%) followed by *P. aeruginosa* (40%), *Klebsiella* spp. (11.6%), CoNS (10.8%) *Protues* spp. (10%), and *E. coli* (6.67%) (Fig. 1). Figure 2 shows the prevalence of bacteria isolated from different types of wound infections in relation to gender. Regarding isolates, 121 isolates (71.2%) were isolated from males. While 49 isolates (28.8%) were isolated from females. The prevalence of *S. aureus* and *E. coli* was greater in males. While the prevalence of CoNS, *P. aeruginosa*, *Klebsiella* spp., and *Proteus* spp., was greater in females.

### Antibiotic sensitivity and resistance profiles of Gram-positive isolates

Both *S. aureus* and CoNS showed high resistance to piperacillin, cefoxitin, ampicillin/sulbactam, and amoxicillin/clavulanic acid. MRSA was detected in 89.9% of *S. aureus* isolates. The details of sensitivity and resistance profiles are mentioned in Table 1. The association between susceptibility or resistance to various antibiotics among *S. aureus* or CoNS was significant in case of rifampin, piperacillin, gentamicin, levofloxacin and ciprofloxacin (*p* < 0.05).

**Table 1:** Antimicrobial sensitivity and resistance profile of Gram-positive isolated from wound and burn infections

Antibiotic	*S. aureus* (N = 75)			CoNS (N = 13)			P value*
	**S%**	I (%)	**R%**	**S%**	I (%)	**R%**	
**Linezolid**	61 (81.3%)	0 (0)	14 (18.6%)	11 (84.6%)	0 (0)	2 (15.4%)	0.777
**Tetracycline**	22 (29.3%)	7 (9.3)	46 (61.3%)	5 (38.5%)	3 (23.1)	5 (38.5%)	0.273
**Chloramphenicol**	52 (69.3%)	14 (18.6)	9 (12%)	11(84.6%)	1 (7.7)	1 (7.7%)	0.554
**Rifampin**	43 (57.3%)	3 (4)	29 (38.7%)	9 (69.2%)	1 (7.7)	3 (23.1%)	**0.018**
**Piperacillin**	1 (1.3%)	0 (0)	74 (98.7%)	2 (15.4%)	0 (0)	11 (84.6%)	**0.010**
**Ampicillin / Sulbactam**	8 (10.7%)	0 (0)	67 (89.3%)	1 (7.7%)	0 (0)	12 (92.3%)	0.744
**Amoxicillin/ Clavulanic**	8 (10.7%)	0 (0)	67 (89.3%)	1 (7.7%)	0 (0)	12 (92.3%)	0.744
**Cefoxitin**	8 (10.7%)	0 (0)	67 (89.3%)	1 (7.7%)	0 (0)	12 (92.3%)	0.744
**Gentamycin**	37 (49.3%)	11(14.7)	27 (36%)	12 (92.3%)	0 (0)	1 (7.7%)	**0.018**
**Levofloxacin**	44 (58.67%)	5 (0.7)	26 (34.67%)	12 (92.3%)	0 (0)	1 (7.7%)	**0.002**
**Ciprofloxacin**	41 (54.67%)	8 (10.7)	26 (34.67%)	12 (92.3%)	0 (0)	1 (7.7%)	**0.030**
**Vancomycin**	64 (85.3%)	0 (0)	11 (14.67%)	11 (84.6%)	0 (0)	2 (15.4%)	0.976

* X^2^ test was used for statistical analysis and P values are significant at < 0.05

### Antibiotic sensitivity and resistance profiles of Gram-negative isolates

Data in Table 2 shows various susceptibility and resistance profiles to different antibiotics. For instance, *P. aeruginosa* was sensitive to ciprofloxacin (79.2%) and highly resistant to cefazolin (100%) and levofloxacin (83.3%). *Klepsiella* spp. were sensitive to gentamicin (78.6%) and highly resistant to cefazolin (100%) and ceftazidime (85%). *Protius* spp. was highly resistant to ampicillin/sulbactam (100%), cefazolin (100%) and ceftazidime (91.9%). Finally, *E. coli* showed absolute resistance against cefuroxime and cefazolin (100%) and high resistance against ampicillin/sulbactam (87.5%). In the case of levofloxacin and ciprofloxacin, the association between susceptibility or resistance to various antibiotics was significant (*p* < 0.05).

**Table 2:** Antimicrobial sensitivity and resistance profile of Gram-negative organisms isolated from wound and burn infections

Antibiotic	*P. aeruginosa* (N = 48)			*Klebsiella* spp. (N = 14)			*Proteus* spp. (N = 12)			*E. coli* (N = 8)			P value*
	**S%**	**I (%)**	**R%**	**S%**	**I (%)**	**R%**	**S%**	**I (%)**	**R%**	**S%**	**I (%)**	**R%**	
**Chloramphenicol**	21 (43.8%)	10 (20.8)	17 (35.4%)	6 (42.9%)	2 (14.3)	6 (42.9%)	8 (66.7%)	3 (25)	1 (8.3%)	5 (62.5%)	0 (0)	3 (37.5%)	0.262
**Tigycyclin**	17 (35.4%)	18 (37.5)	13 (27.1%)	1 (7.1%)	9 (64.3)	4 (28.6%)	4 (33.3%)	8 (66.7)	0 (0%)	2 (25%)	6 (75)	0 (0%)	0.186
**Gentamycin**	29 (60.4%)	3 (6.3)	16 (33.3%)	11 (78.6)	1 (7.1)	2 (14.3%)	6 (50%)	1 (8.3)	5 (41.7%)	4 (50%)	0 (0)	4 (50%)	0.318
**Levofloxacin**	8 (16.7%)	0 (0)	40 (83.3%)	9 (64.3%)	1 (7.1)	4 (28.6%)	7 (58.3%)	1 (8.3)	4 (33.3%)	5 (62.5)	1 (12.5)	2 (25%)	**< 0.0001**
**Ciprofloxacin**	38 (79.2%)	2 (4.2)	8 (16.7%)	6 (42.9%)	4 (28.6)	4 (28.6%)	7 (58.3%)	1 (8.3)	4 (33.3%)	2 (25%)	2 (25)	4 (50%)	**0.042**
**Ampicillin / Sulbactam**	4 (8.3%)	8 (16.7)	36 (75%)	1 (7.1%)	6 (42.9)	7 (50%)	0 (0%)	0 (0)	12 (100%)	0 (0%)	1 (12.5)	7 (87.5%)	0.532
**Amoxicillin/ Clavulanic**	11 (23%)	5 (10.4)	32 (66.7%)	3 (21.4%)	4 (28.6)	7 (50%)	0 (0%)	6 (50)	6 (50%)	0 (0%)	5 (62.5)	3 (37.5%)	0.189
**Ticracillin** **/tazobactam**	15 (31.3%)	15 (31.3)	18 (37.5%)	2 (14.3%)	5 (35.7)	7 (50%)	0 (0%)	6 (50)	6 (50%)	4 (50%)	1 (12.5)	3 (37.5%)	0.090
**Cefuroxime**	9 (18.8%)	9 (18.8)	30 (62.5’%)	3 (21.4%)	2 (14.3)	9 (64.3%)	2 (16.7%)	3 (25)	7 (58.3%)	0 (0%)	0 (0)	8 (100%)	0.497
**Cefazolin**	0 (0%)	0 (0)	48 (100%)	0 (0%)	0 (0)	14 (100%)	0 (0%)	0 (0)	12 (100%)	0 (0%)	0 (0)	8 (100%)	N/A
**Ceftazidime**	6 (12.5%)	7 (14.6)	35 (73%)	2 (14.3%)	0 (0)	12 (85.%)	0 (0%)	1 (8.3)	11 (91.7)	2 (25%)	0 (0)	6 (75%)	0.445
**Imipineme**	24 (50%)	8 (16.7)	16 (33.3%)	7 (50%)	2 (14.3)	5 (35.%)	1 (8.3%)	7 (58.3)	4 (33.3%)	4 (50%)	1 (12.5)	3 (37.5%)	0.472
**Trimethoprim/ sulfamethoxazole**	19 (39.6%)	8 (16.7)	21 (43.8%)	11 (78.6)	0 (0)	3 (21.%)	7 (58.3%)	0 (0)	5 (41.7%)	2 (25%)	0 (0)	6 (75%)	0.053

* X^2^ test was used for statistical analysis and P values are significant at < 0.05

### Gender and age group correlations with the antibiotic resistance profile

Gender was not significantly correlated with the antibiotic resistance profile among Gram-positive samples (67 patients) and Gram-negative samples (53 patients) (Tables 3 and 4, respectively). The correlations between age group and antibiotic resistance profile among Gram-positive and Gram-negative samples were illustrated in Tables 5 and 6, respectively. No statistically significant difference was detected between the resistance profiles of tested antibiotics among both Gram-positive and Gram-negative samples and the participant’s age group (*p* > 0.05).

**Table 3:** Correlation between gender and antibiotic resistance profile among Gram-positive samples

Antibiotic	Resistance profile			P-value*
	Sensitive	Intermediate	Resistant	
**Linezolid**				
Male Female	38 15	0 0	9 5	0.547
**Tetracycline**				
Male Female	9 3	9 0	29 17	0.079
**Chloramphenicol**				
Male Female	31 14	9 3	7 3	0.919
**Rifampin**				
Male Female	25 10	2 2	20 8	0.662
**Piperacillin**				
Male Female	1 0	0 0	46 20	0.511
**Cefoxitin**				
Male Female	3 1	0 0	44 19	0.827
**Gentamycin**				
Male Female	22 8	7 4	18 8	0.828
**Ampicillin/Sulbactam**				
Male Female	3 1	0 0	44 19	0.827
**Levofloxacin**				
Male Female	3 1	0 0	44 19	0.827
**Ciprofloxacin**				
Male Female	25 10	3 2	19 8	0.872
**Amoxicillin/Clavulanic**				
Male Female	3 1	0 0	44 19	0.827
**Vancomycin**				
Male Female	3 1	0 0	44 19	0.827

*Chi square test; P-value was set to 0.05

**Table 4:** Correlation between gender and antibiotic resistance profile among Gram-negative samples

Antibiotic	Resistance profile			P-value*
	Sensitive	Intermediate	Resistant	
**Ticracillin /Tazobactam**				
Male Female	5 0	13 7	23 5	0.168
**Tigycyclin**				
Male Female	9 2	21 5	11 5	0.314
**Chloramphenicol**				
Male Female	17 4	7 1	17 7	0.547
**Cefuroxime**				
Male Female	2 4	6 1	33 7	0.063
**Cefazolin**				
Male Female	0 0	0 0	41 12	N/A
**Imipineme**				
Male Female	12 7	10 0	19 5	0.076
**Gentamycin**				
Male Female	21 5	4 1	16 6	0.794
**Ampicillin/Sulbactam**				
Male Female	3 1	3 2	35 9	0.609
**Amoxicillin/Clavulanic**				
Male Female	3 3	9 0	29 9	0.073
**Ceftazidime**				
Male Female	1 0	5 2	35 10	0.803
**Levofloxacin**				
Male Female	27 8	3 0	11 4	0.601
**Ciprofloxacin**				
Male Female	24 6	4 2	13 4	0.772
**Trimethoprim/Sulfamethoxazole**				
Male Female	11 5	6 3	24 4	0.304

*Chi square test; P-value was set to 0.05; N/A: not applicaple

**Table 5:** Correlation between age group and antibiotic resistance profile among Gram-positive samples

Antibiotic	Resistance profile			P-value*
	Sensitive	Intermediate	Resistant	
**Linezolid**				
1–20 20–40 41–60	25 21 7	0 0 0	10 3 1	0.270
**Tetracycline**				
1–20 20–40 41–60	7 4 1	4 4 1	24 16 6	0.964
**Chloramphenicol**				
1–20 20–40 41–60	23 16 6	8 3 1	4 5 1	0.742
**Rifampin**				
1–20 20–40 41–60	20 12 3	2 1 1	23 11 4	0.804
**Piperacillin**				
1–20 20–40 41–60	1 0 0	0 0 0	34 24 8	0.629
**Cefoxitin**				
1–20 20–40 41–60	4 0 0	0 0 0	31 24 8	0.143
**Gentamycin**				
1–20 20–40 41–60	18 9 3	7 3 1	10 12 4	0.513
**Ampicillin / Sulbactam**				
1–20 20–40 41–60	4 0 0	0 0 0	31 24 8	0.143
**Levofloxacin**				
1–20 20–40 41–60	4 0 0	0 0 0	31 24 8	0.143
**Ciprofloxacin**				
1–20 20–40 41–60	22 9 4	2 2 1	11 13 3	0.400
**Amoxicillin/ Clavulanic**				
1–20 20–40 41–60	4 0 0	0 0 0	31 24 8	0.143
**Vancomycin**				
1–20 20–40 41–60	4 0 0	0 0 0	31 24 8	0.143

*Chi square test; P-value was set to 0.05

**Table 6:** Correlation between age group and antibiotic resistance profile among Gram-negative samples

Antibiotic	Resistance profile			P-value*
	Sensitive	Intermediate	Resistant	
**Ticracillin / Tazobactam**				
1–20 20–40 41–60	2 0 3	13 4 3	8 11 9	0.065
**Tigycyclin**				
1–20 20–40 41–60	3 2 6	11 10 5	9 3 4	0.157
**Chloramphenicol**				
1–20 20–40 41–60	7 7 7	4 2 2	12 6 6	0.838
**Cefuroxime**				
1–20 20–40 41–60	4 0 2	3 3 1	16 12 12	0.448
**Cefazolin**				
1–20 20–40 41–60	0 0 0	0 0 0	23 15 15	N/A
**Imipineme**				
1–20 20–40 41–60	10 4 5	3 2 5	10 9 5	0.369
**Gentamycin**				
1–20 20–40 41–60	12 5 9	2 2 1	9 8 5	0.677
**Ampicillin / Sulbactam**				
1–20 20–40 41–60	3 0 1	4 0 1	16 15 13	0.185
**Amoxicillin/ Clavulanic**				
1–20 20–40 41–60	3 1 2	2 2 5	18 12 8	0.309
**Ceftazidime**				
1–20 20–40 41–60	0 0 1	5 1 1	18 14 13	0.285
**Levofloxacin**				
1–20 20–40 41–60	19 8 8	0 2 1	4 5 6	0.171
**Ciprofloxacin**				
1–20 20–40 41–60	17 5 8	1 4 1	5 6 6	0.077
**Trimethoprim / Sulfamethoxazole**				
1–20 20–40 41–60	6 5 3	5 3 3	12 8 8	0.942

*Chi square test; P-value was set to 0.05; N/A: not applicaple

### MDR, XDR, and PDR profiles of isolates

MDR, XDR, and PDR percentages of each bacteria were calculated out of the total number of isolates of each bacteria. Table 7 shows that 52.4% of total isolates elucidated a MDR profile (i.e. resistance to at least 3 antibiotic classes). The overall MDR of Gram-positive isolate was 50% (56% for *S. aureus* and 15.4% for CoNS). Total MDR rate of Gram-negative isolates was 54.9% with the highest prevalence pattern of *E. coli* (87.5%). For other Gram-negative species, the MDR rates were as follows: *P. aeruginosa* (54.2%), *Klebsiella* spp. (50%), and *Proteus* spp. (41.7%). Both *Proteus* spp. and *E. coli* had significant distributions of resistance profiles across antibiotic classes (*p* < 0.05). Only 4.7% and 2.9% of all isolates showed XDR and PDR profile, respectively. XDR rate of Gram-positive isolate was 2.3% and that of Gram-negative isolates was 7.3%, while the PDR rates of Gram-positive and Gram-negative isolates were 3.4% and 2.4%, respectively.

**Table 7:** Prevalence of MDR, XDR and PDR profile of wound infections isolates

Organism	R0 %	R1 %	R2 %	R3 %	R4 %	R5 %	R6 %	R7 %	R$\ge 8$ %	P value*	MDR	XDR	PDR
***S. aureus*** **(n = 75)**	0 (0%)	3 (4%)	3 (4%)	1 (1.3%)	10 (13.3%)	18 (24%)	7 (9.3%)	8 (10.7%)	25 (33.3%)	0.997	42 (56%)	2 (2.7%)	2 (2.7%)
**CoNS** **(n = 13)**	1 (7.7%)	0 (0%)	0 (0%)	1 (7.7%)	5 (38.5%)	4 (30.8%)	0 (0%)	0 (0%)	2 (15.4%)	0.437	2 (15.4%)	0 (0%)	1 (7.7%)
***P. aeruginosa*** **(n = 48)**	0 (0%)	0 (0%)	3 (6.3%)	7 (14.6%)	5 (10.4%)	6 (12.5%)	6 (12.5%)	4 (8.3%0	16 (33.3%)	0.981	26 54.2%	4 (8.3%)	1 (2.1%)
***Klebsiella*** **spp.** **(n = 14)**	0 (0%)	0 (0%)	0 (0%)	3 (21.4%)	4 (28.6%)	1 (7.1%)	2 (14.3%)	0 (0%)	4 (28.6%)	0.437	7 (50%)	1 (7.1%)	1 (7.1%)
***Protues*** **spp.** **(n = 12)**	0 (0%)	0 (0%)	0 (0%)	0 (0%)	3 (25%)	5 (41.7%)	0 (0%)	0 (0%)	4 (33.3%)	**0.040**	5 (41.7%)	0 (0%)	0 (0%)
***E. coli*** **(n = 8)**	0 (0%)	0 (0%)	0 (0%)	0 (0%)	1 (12.5%)	3 (37.5%)	0 (0%)	0 (0%)	4 (50%)	**0.040**	7 (87.5%)	1 (12.5%)	0 (0%)

*Chi square test; P-value was set to 0.05

R0 = Isolates are sensitive to all antimicrobials tested

Key: R1-R8 = Number of antimicrobial classes in which a given isolate was resistant

MDR = Multi-drug resistance; number of isolates resistant to three or more of antibacterial classes

XDR = Extensive drug resistance; number of isolates resistant to all classes of antibiotics except one class of antibiotic

PDR = Pan drug resistance; number of isolates resistant to all classes of antibiotics

% of MDR, XDR and PDR were calculated out of number of isolates belonging each bacteria

## Discussion

This survey assessed the prevalence of various bacterial species isolated from different wound infections among a group of patients in Upper Egypt. Of the 146 population subjects included in this survey, 120 patients elucidated positive bacterial growth with a high isolation rate of 82.2%. This was approximately similar to the finding of Mohammed et al. who reported an isolation rate of 83.9% for wound infections from inpatients and outpatients with pus and/or wound discharge in Northwest Ethiopia [11]. Our overall isolation rate was relatively higher than that of previous studies conducted in Ethiopia [12, 13]. The difference could be attributed to the etiology of bacterial infection and the source of wound infections from which samples were obtained. Another reason that may explain this variation is the adopted protocol of infection control and antibiotic prophylaxis that may play a crucial role in bacterial growth. Also, the fastidious nature of some bacteria may be responsible for their inability to grow[14].

The prevalence of poly-bacterial species (55%) was higher than that of mono-bacterial species (45%). This was different from the findings of Hassan et al. [15] who reported a higher prevalence of mono-bacterial isolates at 60% and 40% of mixed bacterial species. Likewise, Ares et al. [16] and Bessa et al. [2] reported a predominance of mono-microbial infections over poly-microbial infections with rates of 88.6% and 72.8%, respectively. The presence of monotype or poly-microbial communities of bacteria has a multifactorial nature. For instance, the wound state, microbial density, previous treatment of the wound, dermal moisture, and nutrient availability [17] could explain the difference.

Our findings revealed almost similar proportions of Gram-positive bacteria (51.8%) and Gram-negative strains (48.2%). This was in line with Ares et al. [16] and Bessa et al. [2]. On the other hand, some prior studies showed a predominance of Gram-negative isolates over Gram-positive strains [15, 17, 18]. The diversity of results could be related to the variations in participants’ demographic characteristics. Moreover, nosocomial infection increases the prevalence of MDR bacteria [15].

Our findings revealed that *S. aureus* and *P. aeruginosa* were the dominant pathogens. This was consistent with the findings of Puca et al. [19]. *S. aureus* represented 62.5% of all isolates and 85.2% of isolated Gram-positive strains that have been isolated from all samples of different wound infections. This was comparable to the findings of Ahmed et al. [20] in a study conducted in Upper Egypt. They stated that *S. aureus* was identified in 61% of specimens, surgical site infections, abscesses, and burn infections were the most common sites of *S. aureus* isolates representing 59%, 56%, and 52%, respectively. This was to some extent in agreement with the present results. The prevalence of *S. aureus* was 65%, which coincided with the prevalence reported by Mulu et al. [21] among 151 wound swabs. In like manner, different studies confirmed the predominance of *S. aureus* isolated from wound infections [14, 22, 23]. This is not surprising because *S. aureus* is the common skin commensal. Also, exogenous or endogenous infections could be the source of the *S. aureus* infection.

CoNS accounted for 10.8% of all isolates which was comparable to a retrospective study conducted in intensive care unit of Ain Shams University Hospitals in Egypt, which reported a rate of 12.5% from different infection sites [3]. Another study conducted in Ethiopia showed CNS prevalence of 14.5%, which is relatively higher than our findings [24].

Among all isolates, *P. aeruginosa* was the second pathogen that was isolated from wound infections, with a prevalence of 40%. This prevalence was compatible with that recorded by Manikandan and Amsath [25]. Another Gram-negative species prevalence was for *Klebsiella* spp. (11.6%), *Proteus spp*. (10%), and *E. coli* (6.7%). A previous study in Egypt revealed a predominance of *Klebsiella* spp. followed by *E. coli* [3]. Another study demonstrated the dominance of *E. coli* among all identified Gram-negative species, followed by *Proteus* spp., with the least prevalence of *P. aeruginosa* [24]. The diversity among different studies could be attributed to the number of participants, environmental factors, providing health services, and individual health care conditions [26].

Beta-lactam ring-based antibiotics had the highest resistance profile to Gram-positive strains, with a prevalence ranging from 84.6 to 98.7%. Our findings revealed a remarkable increase in the prevalence of MRSA compared to a previous survey that was conducted by Ahmed et al. [27] who reported a 24% prevalence of MRSA in the same hospital eight years ago. This raises the alarm about the escalating and noticeable increase in the prevalence of MRSA in Egypt. Another study carried out in Ghana [28] confirmed absolute resistance to oxacillin. *S. aureus* was sensitive to vancomycin with a rate of 85.2%, while vancomycin-resistant *S. aureus* (VRSA) was detected in 14.8% of cases. This was relatively lower than that concluded in Egypt by Ghoniem et al. [29] who reported that the ratio of VRSA was 20.7%. On the other hand, another study has been held in Egypt by Amr et al. [30] demonstrated a lower prevalence of VRSA (8.8%). These discrepancies could be related to the variations in the bacterial culture method and the different geographical settings of these studies.

Regarding Gram-negative sensitivity, All Gram-negative isolates tested positive for absolute resistance to the cefazolin antibiotic in our study. *P. aeruginosa* was the most commonly isolated Gram-negative species, with resistance rates ranging from 62.5 to 100% to the ß-lactam antibiotic. Similarly, *P. aeruginosa* had a high prevalence of levofloxacin resistance (83.3%). These findings were quite similar to Nikokar et al. [31] results of the resistance profile rates of *P. aeruginosa* isolated from burn infections and post-surgical sites and wound infection against the cephalosporin antibiotics cefazolin (83.7%) and ceftazidime (68.8%), gentamicin (37.2%), and imipenem (23.3%). While they confirmed a high resistance rate against ciprofloxacin (66.3%), our findings supported the lowest resistance being against ciprofloxacin (16.7%).

*Klebsiella* spp. exhibited high resistance to ß-lactam antibiotics. This was in agreement with previous literature conducted in Bangladesh [7]. This was consistent with the conclusion of a review conducted in Asia [32] that highlighted the rising danger of MDR of *Klebsiella* spp. Antimicrobial resistance as a result of antibiotic misuse is a life threatening issue affecting both males and females of all ages without regard to certain genders or age groups [33]. The current study confirmed that idea, as no difference was detected regarding associating patients’ gender and age groups with the resistance profile of the tested antimicrobials against Gram-positive and Gram-negative isolates.

The prevalence of the MDR profile was slightly higher among the Gram-negative isolates compared to Gram-positive species. The MDR of Gram-negative rate was 54.9%, which is close to the MDR rates reported in two studies conducted in Ethiopia. The first study reported an MDR prevalence of 51% among bacteria isolated from open fracture wounds [34]. The second study found that 59.3% of bacteria from wound infections had an MDR profile [35]. Relatively similar results were published in Egypt, which recorded 73.9% and 70% MDRs of *E. coli* and *Pseudomonas* spp., respectively, against the majority of tested antibiotics. Regarding Gram-positive isolates, the overall MDR was 50%, with a higher MDR profile for *S. aureus* (56%) followed by CoNS (15.4%). This was less than the findings of Melake et al. who found that the prevalence of MDR of *S. aureus* and CoNS isolated from burn wounds in Egypt were 73.5% and 47%, respectively.

The main limitations of the current study can be summarized as follows; (1) the use of a disk-diffusion approach to detect the bacterial susceptibility, which has relative reliability, (2) the generalization of the study findings has to be interpreted with caution as possible due to heterogeneity of subjects and settings in different geographic areas; and (3) the availability of anaerobic bacteria was not accounted for in the current study due to the shortage in the culture conditions.

## Conclusions

Within the limitations of the current findings, it can be concluded that there was a dominance of polymicrobial wound samples. *S. aureus* and *P. aeruginosa* were the most commonly isolated pathogens. A high rate of MRSA was revealed among *S. aureus* isolates. Vancomycin and linezolid were the most effective drugs against *S. aureus* and ciprofloxacin was the most effective one against *P. aeruginosa*. Several isolates elucidated MDR profile for all tested classes of antibiotics, which indicates a serious exacerbation of bacterial resistance and a difficulty finding treatment options for all infections.

## Data Availability

All data generated or analyzed during this study are included in this published article.
